# Support From Parents, Peers, and Teachers Is Differently Associated With Middle School Students’ Well-Being

**DOI:** 10.3389/fpsyg.2021.758226

**Published:** 2021-12-02

**Authors:** Frances Hoferichter, Stefan Kulakow, Miriam C. Hufenbach

**Affiliations:** ^1^Department of School Pedagogy, University of Greifswald, Greifswald, Germany; ^2^Department of Psychology, University of Potsdam, Potsdam, Germany

**Keywords:** social support, teachers, peers, parents, middle school students, well-being

## Abstract

Parents, peers, and teachers provide a powerful context for school students’ well-being. However, a detailed and systematic analysis of how parental, peer, and teacher support relate to students’ well-being, measured by the dimensions self-worth, psychological and physical well-being, is still missing. To address this research gap, the following study investigates 733 adolescent German students from grades 7 and 8 (M*_*age*_* = 13.97, *SD* = 0.41, 52% girls) with respect to their perceived supportive relationships at home and within the school context. The study considers gender, socioeconomic status, and school form as potential confounders. The results of the structural equation model, analyzed with the statistical software R, indicate that perceived teacher support was positively related to students’ self-worth and physical well-being, while peer support was related to psychological well-being. Students who perceived their parents as supportive reported higher well-being with respect to all three dimensions investigated.

## Introduction

Research on school students’ well-being has gained increasing attention over the last decade as a response to the drastic increase in mental health problems, referred to as the “millennial morbidity,” among school students from developed countries (Palfrey et al., 2005; Suhrcke et al., 2008; p. 43). Well-being is a key factor to address health concerns of school students, as well-being is associated with a wide range of positive outcomes related to health and academia (Amholt et al., 2020). In particular, well-being is associated with educational attainment, academic success (Suldo et al., 2011; Simovska et al., 2016), low levels of burnout and depressive symptoms (Upadyaya and Salmela-Aro, 2013), decreased test anxiety (Steinmayr et al., 2016), and a minimized risk of psychopathology (Pynoos et al., 1999). When investigating school students’ well-being, the immediate social environment should be considered, as an individuals’ well-being is closely linked to the quality of her or his social relationships (Umberson and Montez, 2010). However, thus far, little systematic research has been conducted on the association between school students’ well-being and their immediate social environment, including parents, peers, and teachers. On a micro-level, students frequently interact with their parents, mostly at home, and with their peers and teachers inside and outside school, while parents, teachers, and peers in turn interact with each other on a meso-level (see ecological systems theory by Bronfenbrenner, 1979). Following this approach, the conceptual model of well-being in schools (Konu and Rimpelä, 2002) emphasizes the importance of students’ surroundings and community for students’ well-being. To conceptualize well-being in the current study, we follow the approach by Ravens-Sieberer et al. (2010) and define well-being as a three-dimensional concept, including a persons’ physical and psychological state as well as his or her self-worth (Ravens-Sieberer et al., 2014). Physical well-being describes a person’s fitness and energy levels, as well as their levels of physical complaints and malaise. Psychological well-being refers to life satisfaction, including positive emotions and the absence of feelings of sadness and loneliness. As a third dimension, self-worth covers the value an individual assigns to himself or herself and feelings of contentment with oneself. While the association between well-being and late adolescents’ health and life satisfaction is well researched, studies that systematically investigate the role of supportive relationships with parents, teachers, and peers among middle school students are underrepresented. To address this research gap, this study was designed to investigate the association between parental, teacher, and peer support and middle school students’ well-being.

## Social Support and Well-Being

In general, social support refers to the social structure in which an individual is embedded, including specific aspects served by interpersonal relationships (Taylor, 2011). These aspects include, for example, feeling part of a social network, being engaged in bidirectional relationships characterized by close ties, mutual care, and esteem, or receiving help if needed (Sarason and Sarason, 2009). Due to the beneficial nature of social support with respect to mental and physical health, self-worth, self-esteem, academic success, and life satisfaction, various research disciplines have established theories built around social support. Among the most prominent is the buffering hypothesis (Cohen and Wills, 1985), according to which social support mitigates feelings of stress, as social support presents an available resource to cope with stressors (Kikusui et al., 2006). Moreover, by taking advantage of social support in stressful situations, further resources can be gained that help overcome stressors (Conservation of Resources Theory, Hobfoll et al., 1990). Following the direct effect theory (Hashimoto et al., 1999), social support is also beneficial in the absence of stress and serves to increase individuals’ well-being. Research on developmental aspects of children and youth within the frameworks of social attachment (Bowlby, 1982; Shaver and Mikulincer, 2010) and the need-to-belong theory (Baumeister and Leary, 1995) calls attention to the instinctive psychological need to experience integration, membership, mutual trust, and safety (Furman, 1998). These are important antecedents of personal growth, cognitive and behavioral skills, and, above all, well-being (Karreman and Vingerhoets, 2012). Although these theories and models approach social relationships differently, focusing on specific aspects of social relationships, they commonly acknowledge the beneficial role of social relationships for individuals’ well-being.

However, social support may be most beneficial if it meets the needs of an individual (cf. Ozbay et al., 2007; Sarason and Sarason, 2009; de Grey et al., 2018). Based on this premise, school students may have different needs with respect to the support they perceive from their immediate environment. Hence, parental, peer, and teacher support may be differently associated with students’ self-worth, psychological and physical well-being.

## Perceived Social Support and Middle School Students’ Well-Being

Thus far, empirical studies on the association between the three dimensions of well-being and social support from parents, teachers, and peers have either investigated single dimensions of well-being (e.g., in parent-child research) or used well-being as an umbrella concept for behavioral, cognitive, or socio-emotional outcomes of school students. Therefore, the impression is given that social support in general—no matter from whom—presents a common remedy to enhance school students’ overall well-being. However, systematic empirical research on the different sources from which students receive their support is still missing. For the sake of students’ self-worth, mental health, and physical health, it is essential to identify which sources of support relate to the three dimensions of well-being to shape the network of parent-child, teacher-student, and peer relationships inside and outside school.

### Parental Support

Empirical research commonly emphasizes the positive link between parental support and students’ well-being. Various studies—using small samples—have found that parental support is linked to children’s psychological well-being. Such research includes studies with 128 late adolescents (Gecas and Schwalbe, 1986), 177 late adolescents (Xiaoyu et al., 2019), and 554 middle adolescent students (Francis et al., 2020). Similarly, students’ physical well-being, which has often been assessed by students’ physical health, is closely linked to parental support. For example, Wickrama et al. (1997) found that parental support (perceived and observed) had direct and indirect effects on adolescents’ physical health. In particular, changes in physical complaints could be explained through the level of parental support; in other words, physical complaints increased among children with low-to-medium parental support (Wickrama et al., 1997). Other studies have found similar results; for example, middle school students exhibited better physical well-being if they had parents who cared for them (Jin et al., 2020). In a representative longitudinal study with adults aged 25–74 years, Shaw et al. (2004) found that those adults who reported a lack of parental support during their childhood were more likely to develop chronic health conditions and depressive symptoms in adulthood (Shaw et al., 2004). Likewise, various studies indicate a perceived deficiency of parental support to be related to low levels of physical and mental health, such as increased internalized and externalized problems (Berber and Harmon, 2002), internalized distress (Costa et al., 2015), and impaired psychological functioning (Inguglia et al., 2018).

With respect to social support and students’ self-worth, studies are limited to very small sample sizes and specific cohorts. For example, a study with 38 early adolescent students (7–12 years of age) who had experienced domestic violence found that maternal support and peer support were more strongly related to students’ self-worth than support from their teachers (Riesen and Porath, 2004). In another study with 100 adolescents aged 13–18 from Malaysia, researchers suggest that parental and teacher support were not significantly related to students’ self-worth, but peer support was related to improved self-worth (Chii et al., 2017).

In sum, empirical studies have found that parental support constitutes an essential component to enhance students’ self-worth, psychological well-being, and physical well-being (for an overview, see Cripps and Zyromski, 2009; Thomas et al., 2017).

### Peer Support

Most empirical findings suggest that parental support is positively linked to psychological and physical well-being and self-worth; however, the role of support from peers with respect to the three dimensions of well-being is not as clear. Thus far, research on peer support has focused primarily on students’ behavioral, socio-emotional, and health outcomes linked to an overall framework of well-being. Peer support has been investigated primarily in educational research, where well-being has often been used as a flexible term that includes, for example, socio-emotional components, satisfaction, health, positive emotions, and the absence of worries and conflicting relationships at school (Hascher, 2003; Rathmann et al., 2018; Hoferichter et al., 2020). Therefore, the impression arises that many studies apply their own unique definition of well-being, which leads to the common perception that peer support in general contributes to students’ overall well-being. It is clear that students’ relationships at school decisively determine whether students experience positive emotions at school, feel a sense of belonging, are satisfied with school, and exhibit greater mental and physical health; however, peer support has not yet been investigated systematically with respect to school students’ self-worth, psychological, and physical well-being.

Focusing on the role of peers within the school context, peer support has been shown to be related to low levels of test anxiety (Hoferichter and Raufelder, 2015), lower levels of depressive symptoms and loneliness (Holt et al., 2018), higher school satisfaction (Verkuyten and Thijs, 2002), and higher self-worth (Harter, 1999; Adams et al., 2011) among school students. In particular, students’ friendships with peers at school were found to be relevant for students’ self-worth (Maunder and Monks, 2019). In contrast, peer competition at school was related to low scholastic well-being (Hoferichter and Raufelder, 2017), and the experience of peer victimization and bullying was related to poor mental health and impaired overall well-being (Rigby, 2000; Rivers et al., 2009; Arslan et al., 2021).

### Teacher Support

Investigations on how teacher support relates to physical, psychological well-being and self-worth of students are rare. The few studies that are available on the topic found that perceived teacher support was associated with higher psychological well-being (Tennant et al., 2014) and physical well-being (Hoferichter and Raufelder, 2021) but was less likely significantly related to students’ self-worth (Riesen and Porath, 2004; Ozier, 2008).

Students spend a large part of their time at school where they are accompanied and supported by teachers. Thereby, supportive teachers may act as mentors, provide strengths-based feedback, support students’ personal and academic success, treat them fairly and with appreciation. In short, teachers present a major socialization unit and therefore it stands to reason that teachers’ support relates to how students feel and think about themselves. Investigating the unique relationship between teachers’ support next to parental and peer support promises to shed light on the complex mechanisms of social support and students’ well-being.

## Aims and Exploratory Approach

This study aims to investigate the association between perceived parental, peer, and teacher support and middle school students’ psychological and physical well-being and self-worth. By systematically analyzing the three major sources of support in school students’ daily lives inside and outside school, this study contributes to research on well-being in relation to social support.

To consider major confounding factors with respect to students’ well-being, we included gender, socioeconomic status, and school form in the investigation. In general, girls tend to report lower levels of psychological functioning, psychological and physical health, and self-worth compared with boys (Thomas and Daubman, 2001; Phares et al., 2004; Savoye et al., 2015).

Besides gender, socioeconomic status (SES) has been shown to play an essential role for students’ psychological and physical well-being, self-confidence, and self-esteem (Twenge and Campbell, 2002; Marcen et al., 2013; Sweeting and Hunt, 2014; Fassbender and Leyendecker, 2018). In detail, a higher education, financial resources, and accordingly, a higher SES positively relate to well-being, which was indicated in studies investigating both objective measures of SES (e.g., education, household and personal income) as well as subjective (e.g., financial strain) (Wang et al., 2010). Thereby, higher SES is associated with less daily hassles, less depression and a higher life satisfaction (Fassbender and Leyendecker, 2018). School form was considered in the analysis, as school students in Germany, where this study was conducted, attend different schools according to their abilities, interests, and future career plans, which may impact their well-being differently. Three major school forms can be distinguished: lower-track schools (which finish with grade 9 or 10), higher-track schools (which finish with grade 12 and certify students to attend university), and mixed-track schools (where various school leaving exams can be undertaken, and classes go up to grade 13). As the school tracks vary with respect to school culture, future career perspectives, and academic demands, students from higher-track schools have been shown to exhibit different levels of exhaustion from schoolwork (Salmela-Aro et al., 2008), perceived stress (Kulakow et al., 2021), and school satisfaction (Van Houtte, 2006, 2017; Geven, 2019) compared with students from lower-track schools.

In conclusion, empirical studies suggest that parental support is associated with students’ self-worth and psychological and mental health and therefore presents a foundation for well-being. Usually, well-being was framed as overall well-being, scholastic well-being, or socio-emotional well-being. Hence, studies within the school context have applied various definitions of well-being and have not systematically investigated parental, peer, and teacher support with respect to students’ self-worth, psychological and physical well-being.

In general, we expect positive relationships between the three sources of support and the dimensions of well-being, as suggested by various theoretical frameworks stated above. However, as empirical findings on the association between parental, peer, and teacher support with psychological and physical well-being and self-worth among healthy middle school students have not been tested within one statistical model yet and because previous studies in the field reveal inconsistent results, we follow an exploratory approach. Hence, we investigate how parental, peer, and teacher support each relate to physical, psychological well-being and self-worth, respectively. As parents, peers, and teachers act as different socializers and as such have different methods of socialization, their support may be associated differently to students’ dimensions of well-being. While parents are the primer source of socialization and act as role models (Eisenberg et al., 1998), they might provide support as they want their child to succeed and feel happy. In turn teachers’ support is limited to the school context where teachers support their students succeed academically. Thereby, teachers might see the support of students as part of their teaching profession. When it comes to peers, they might give support to others within their peer group, particularly if a peer member feels sad or discouraged.

## Materials and Methods

### Participants and Procedure

The sample included 733 adolescent German students from grades 7 and 8 (*M*_*age*_ = 13.97, *SD* = 0.41, 52% girls) in the federal state of Mecklenburg-Vorpommern. The students came from 11 randomly chosen secondary schools. As the German school system allocates students to educational tracks, the three typical educational tracks were included in the sample: three low-track schools (*n_*students*_* = 192), five high-track schools (*n_*students*_* = 442), and two mixed-track schools (*n_*students*_* = 99). Students were surveyed using a questionnaire during the winter term of the German school year 2018–2019. As there is only a small proportion of ethnic diversity in Mecklenburg-Vorpommern (4.3%; Statistical Office Mecklenburg-Western Pomerania, 2018), data on ethnic background was not gathered since it could have impaired the anonymity of the sample.

To ensure ethically sound research practices (American Psychological Association, 2002), a strict procedure for the collection of data was followed. First, permission to conduct the study was obtained from the educational authorities (Ministry for Education, Science and Culture, Mecklenburg-Western Pomerania). Second, schools were informed about the nature and procedure of the study and were asked to participate. Third, parents and students were approached and asked to provide their written consent. On the day of data collection, at least two trained research assistants highlighted again the nature of the study and ensured the anonymity of data collection. They explained the use of the survey instrument, particularly the Likert scales, and answered questions about the study or ambiguous items if necessary.

### Measures

#### Well-Being

To measure different aspects of well-being, three subscales of the Kid-KINDL-R (Ravens-Sieberer and Bullinger, 2000) were used. The Kid-KINDL-R is a self-report questionnaire to survey health-related quality of life, suitable for both healthy and clinical populations. The subscale *physical well-being* consists of four statements pertaining to the participants’ experience of physical health during the last week. Participants rated the frequency of the described sensation, such as “I have felt sick” or “I had a lot of strength and stamina,” on a 5-point Likert scale from 1 (“never”) to 5 (“always”). As three of the four items are negative, they were recoded before analysis. Based on the current sample, the subscale showed an internal consistency of α = 0.76.

The subscale *psychological well-being* consists of four statements pertaining to the participants’ mental state during the last week. Participants rated the frequency of the described emotions, such as “I was afraid” or “I laughed and had a lot of fun,” on a 5-point Likert scale from 1 (“never”) to 5 (“always”). As three of the four items were negative, they were recoded before analysis. Based on the current study, the subscale showed an internal consistency of α = 0.72.

The subscale *self-worth* consists of four statements pertaining to the participants’ thoughts about themselves during the last week. Participants rated the frequency of statements such as “I was proud of myself” or “I had a lot of good ideas” on a 5-point Likert scale from 1 (“never”) to 5 (“always”). Based on the current study, the subscale had an internal consistency of α = 0.85.

#### Parental Support

Parental support was measured using the subscale *support and sympathy* of a questionnaire designed to investigate school-related parental behavior (Reitzle et al., 2001). This subscale consists of four statements, such as “My parents are there for me when I need them.” Participants rate their agreement with those statements on a 5-point Likert scale from 1 (“don’t agree at all”) to 5 (“agree completely”). Based on the current study, the subscale showed an internal consistency of α = 0.73.

#### Teacher and Peer Support

For teacher and peer support, the two subscales *teacher support* and *peer support* of the Teacher and Classmate Support scale were used to measures school-related social support from teachers and classmates of the participants (Torsheim et al., 2000).

The subscale *teacher support* consists of four statements and covers both instructional and emotional support, such as “When I need additional help, I receive it” or “My teachers are interested in me as a person.” and asks the participants to rate their agreement with those statements on a 5-point Likert scale from 1 (“don’t agree at all”) to 5 (“agree completely”). Based on the current study, the subscale showed an internal consistency of α = 0.70.

The subscale *peer support* consists of four statements related to peer acceptance and mutual support within class, such as “Most of my fellow students are friendly and ready to help” or “When a student in my class feels bad, someone in the class tries to help him/her.” and asks the participants to rate these statements on a 5-point Likert scale from 1 (“don’t agree at all”) to 5 (“agree completely”). Based on the current study, the subscale showed an internal consistency of α = 0.78.

#### Covariates

Additional variables were included in the model to rule out spurious associations in the interplay of the variables. Thus, a proxy for socioeconomic status was included that asked about the number of books available in the student’s household (“How many books do you have at home?”; Nachtigall and Kröhne, 2004). Answers were measured on a 5-point Likert scale ranging from 1 (“none or very few”) to 5 (“more than 200”). Moreover, students’ gender was included (0 = female, 1 = male). Lastly, as the German school system consists of various secondary education tracks, the educational tracks (i.e., low-tracking, mixed-tracking, and high-tracking schools) were dummy coded and included as covariates.

### Statistical Analysis

The analyses of the present study were conducted with the free software *R* 4.0 (R Core Team, 2020). Descriptive statistics were computed using the *misty* package (Yanagida, 2020), whereas the inferential analyses were conducted with the *lavaan* package (Rosseel, 2012). All models were specified with the MLR estimator which takes into account potential non-normality as well as non-independence of observations which emerge due to the clustered nature of our sample (Muthén and Muthén, 1998–2017). Additionally, this multilevel structure (students nested in classes) was accounted for using the *cluster* argument of lavaan that adjusts standard errors of the estimates (Asparouhov, 2005).

As in many large samples, the present study was subject to a certain degree of missing data. Of the 733 cases in the study, 104 cases (14.19%) were affected by missingness. Overall, 2.04% of unique values were missing. Thereby, the degree of missingness across all indicators and all cases ranged between 0 and 3.68%. Missing data were accounted for using *full information maximum likelihood* (FIML) estimation under the *missing at random* assumption (Rubin, 1987). FIML is regarded as one of the state-of-the-art techniques for handling missing data (Graham, 2009) that counteracts bias in the parameters which would emerge from more conventional procedures, such as mean imputation or listwise deletion. A missing data analysis revealed two central missing data patterns. The first missing data pattern (*n* = 14) was comprised of students who did not provide information regarding their SES. The second missing data pattern (*n* = 7) resulted from students who did not provide information with regards the support and the well-being scales. Unfortunately, we could not identify auxiliary variables that explained the data loss. However, as the percentage of missing values was so low and Schafer and Graham (2002) indicated that only minor bias would be introduced if an unmeasured variable (which was only moderately correlated with the response) was responsible for the missingness, we decided to use FIML, as the alternatives (e.g., listwise deletion) would rather amplify potential bias.

In a first step, confirmatory factor analyses were conducted to specify the latent variables and to examine the initial measurement model. To explore the theorized relationships between well-being and environmental support, a structural equation model was subsequently specified using the “SEM” function of the *R* package *lavaan* (Rosseel, 2012). In this model, the well-being variables (i.e., psychological well-being, physical well-being, self-worth) were regressed on the predictor variables (i.e., peer support, teacher support, parental support). To control for potential confounds, gender, SES, and school type were additionally specified as predictor variables. The three aspects of well-being were regressed on the control variables, respectively.

#### Bivariate Correlations

Table 1 exhibits all descriptive statistics (i.e., mean, standard deviation, range, skewness, kurtosis) and the manifest correlations of the variables of interest. The correlation matrix revealed highly significant correlations between all variables of interest.

**TABLE 1:** Intercorrelations and descriptive measures.

	**2**	**3**	**4**	**5**	**6**	**7**	**8**	***M***	***SD***	**Range**	**Skewness**	**Kurtosis**
1. Parental support	0.19^***^	0.25^***^	0.24^***^	0.34^***^	0.31^***^	0.14^***^	0.03	3.30	0.57	1–5	–0.89	0.52
2. Teacher support		0.40^***^	0.24^***^	0.20^***^	0.20^***^	0.05	–0.03	3.60	0.66	1–5	–0.67	1.12
3. Peer support			0.22^***^	0.32^***^	0.15^***^	0.13^***^	−0.10^**^	3.96	0.72	1–5	–1.07	1.68
4. Physical well-being				0.62^***^	0.42^***^	0.11^**^	0.19^***^	3.52	0.90	1–5	–0.38	–0.57
5. Psychological well-being					0.48^***^	0.12^**^	0.16^***^	3.80	0.73	1–5	–0.84	0.42
6. Self-worth						0.17^***^	0.17^***^	3.08	0.85	1–5	–0.26	–0.08
7. SES							−0.07^*^	3.38	1.32	1–5	0.09	–0.61
8. Gender								0.48	0.50	0–1	–0.72	–2.00

All measures are standardized. *p < 0.05, **p < 0.01, ***p < 0.001.

#### Structural Equation Model

**FIGURE 1:**
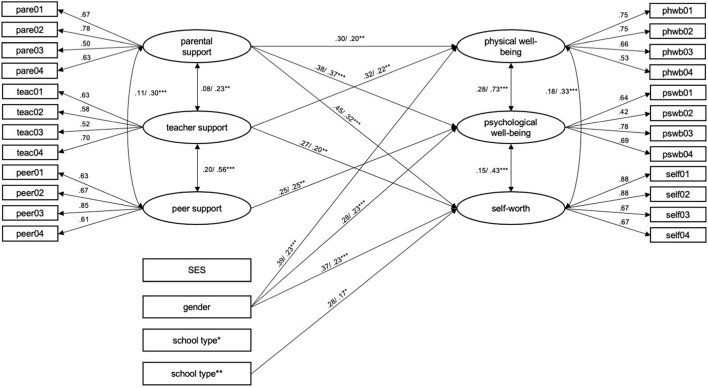
Structural equation model for regression analysis, including control variables. Gender: female = 0, male = 1, school type* = contrasts students from low-track and high-track schools with students from mixed-track schools; school type** = contrasts students from low-track and mixed track school with students from high-track schools; coefficients are displayed unstandardized first, and standardized in second position; only significant paths are shown for clarity; *p < 0.05, **p < 0.01, ***p < 0.001.

The specified SEM achieved an adequate fit to our dataset [χ^2^(321) = 758.99, *p* < 0.001, CFI = 0.91, SRMR = 0.061, RMSEA (90% *CI*) = 0.044 (0.040–0.048)]. As can be seen in Figure 1, the standardized factor loadings of all latent variables ranged between λ_*min*_ = 0.42 and λ_*max*_ = 0.88, indicating a reliable measurement of the variables. This model also included correlations between the predictor variables: Parental support was positively associated with teacher support (*r* = 0.23, *p* < 0.01) and with peer support (*r* = 0.30, *p* < 0.001). Moreover, peer and teacher support were positively associated (*r* = 0.56, *p* < 0.001). The residual correlations of the dependent variables were also significantly associated: psychological well-being was positively associated with physical well-being (*r* = 0.73, *p* < 0.001), and self-worth (*r* = 0.43, *p* < 0.001). Likewise, physical well-being and self-worth were significantly associated (*r* = 0.33, *p* < 0.001).

As can be seen Table 2 and Figure 1, parental support predicted all three constructs of well-being in our dataset: physical well-being (*B* = 0.30, β = 0.20, *SE* = 0.10, *p* < 0.01), psychological well-being (*B* = 0.38, β = 0.37, *SE* = 0.07, *p* < 0.001) and self-worth (*B* = 0.45, β = 0.32, *SE* = 0.08, *p* < 0.001). These significant effects indicate that students who report high support by their parents also report high values of physical well-being, psychological well-being, and self-worth.

**TABLE 2:** Parental support, teacher support, and peer support in relation to physical well-being, psychological well-being, and self-worth.

	**Physical well-being**				**Psychological well-being**				**Self-worth**			
	***Est.***	***SE***	***p***	**β**	***B***	***SE***	***p***	**β**	***B***	***SE***	***p***	**β**
**Predictors**												
Parental support	**0.30**	**0.07**	** < 0.001**	**0.20**	**0.38**	**0.06**	** < 0.001**	**0.37**	**0.45**	**0.07**	** < 0.001**	**0.32**
Teacher support	**0.32**	**0.10**	** < 0.01**	**0.22**	0.02	0.06	0.74	0.02	**0.27**	**0.08**	** < 0.01**	**0.20**
Peer support	0.13	0.09	0.15	0.09	**0.25**	**0.07**	** < 0.001**	**0.25**	–0.12	0.08	0.13	–0.09
SES	**0.06**	**0.03**	** < 0.05**	**0.09**	0.02	0.02	0.40	0.04	0.04	0.03	0.08	0.07
Gender (0 = female, 1 = male)	**0.39**	**0.07**	** < 0.001**	**0.23**	**0.28**	**0.05**	** < 0.001**	**0.23**	**0.37**	**0.06**	** < 0.001**	**0.23**
School type^*^	0.01	0.12	0.91	0.01	–0.16	0.08	0.05	–0.09	–0.07	0.10	0.50	–0.03
School type^**^	0.01	0.09	0.96	0.00	0.10	0.06	0.13	0.08	**0.28**	**0.08**	** < 0.001**	**0.16**
*R* ^2^				0.20				0.33				0.23

Significant results are printed in bold at the p < 0.05 level; *contrasts students from low and high-tracking schools (0) against students from mixed-tracking schools; **contrasts students from low and mixed-tracking schools (0) against students from high-tracking schools (1).

Peer support predicted psychological well-being (*B* = 0.25, β = 0.25, *SE* = 0.08, *p* < 0.01), meaning that students who feel supported by their peers are more likely to feel psychologically well.

Lastly, teacher support significantly predicted physical well-being (*B* = 0.32, β = 0.22, *SE* = 0.11, *p* < 0.01) and self-worth (*B* = 0.27, β = 0.20, *SE* = 0.08, *p* < 0.01). Accordingly, students who received high levels of teacher support reported higher physical well-being and self-worth.

Regarding the effects of the covariates used in this study, we could identify gender differences: Male students reported higher values for physical well-being (*B* = 0.39, β = 0.23, *SE* = 0.07, *p* < 0.001), psychological well-being (*B* = 0.28, β = 0.23, *SE* = 0.05, *p* < 0.001) and self-worth (*B* = 0.37, β = 0.23, *SE* = 0.07, *p* < 0.001) than girls. Additionally, disparities with regards to school types applied: Students from higher-track schools reported significantly higher values of self-worth than students from lower and mixed-track schools (*B* = 0.28, β = 0.17, *SE* = 0.11, *p* < 0.05).

## Discussion

This study investigated how perceived support from parents, peers, and teachers relates to middle school students’ well-being, including self-worth, psychological well-being, and physical well-being, considering gender, socioeconomic status, and school form as confounders.

Considering parental support, the results of the study suggest that students who perceive parental support are more likely to report higher self-worth, psychological well-being, and physical well-being. These results confirm earlier findings by Gecas and Schwalbe (1986); Xiaoyu et al. (2019), and Francis et al. (2020), who investigated small samples of students with respect to parental support and psychological well-being. Students’ physical health has also been found to be related to parental support in various empirical studies conducted by Wickrama et al. (1997), Shaw et al. (2004) and Raufelder et al. (2015). Thus far, research on students’ self-worth associated with parental support has been limited, and results have been inconsistent (Riesen and Porath, 2004; Chii et al., 2017). However, there have been some empirical studies investigating students’ self-esteem, which describes the self-evaluation of ones’ worthiness (Bandura, 1979). Our results are in line with studies investigating students’ self-esteem, indicating that parental support is related to higher self-esteem in children and youth (Birndorf et al., 2005; Boudreault-Bouchard et al., 2013; Perron, 2013). In sum, the current research emphasizes the importance of parental support for all three dimensions of student’s well-being. These findings are particularly important since the investigated cohort of middle school students is in the process of transitioning from childhood to adolescence; this transition implies physical, cognitive, and emotional changes (Eccles, 1999), which may lead to a phase of “storm and stress” and a shift in identity (Twenge and Campbell, 2001). For some students, this phase is related to disrupted relationships with their parents (Erikson, 1963; Arnett, 1999); a decrease in their self-acceptance (Gómez-López et al., 2019), self-esteem (Harter, 1999), mental health, and emotional well-being; and an increase in somatic complaints and fatigue (for an overview, see Viner, 2015).

Next to parent-child relationships, peer relationships become more pronounced during adolescence (Gray et al., 2017), which is reflected in the positive link found between peer support and students’ psychological well-being. These findings indicate that peers present an important context for whether students experience psychological well-being conceptualized through joy, belongingness, and the absence of anxiety and boredom (Ravens-Sieberer and Bullinger, 2000). This finding is in line with other studies that have found that students’ relationships with peers contribute to students’ mental health, subjective well-being (Holt et al., 2018; Moore et al., 2018), scholastic well-being (Hoferichter and Raufelder, 2017), and decreased worry and emotionality related to test anxiety (Hoferichter et al., 2015; Hoferichter and Raufelder, 2015). As such, peer relationships are essential for students’ psychological well-being. Moreover, the quality of these relationships has been shown to determine the neurobiological functioning of students’ brains (Hsu et al., 2013; Landstedt and Persson, 2014; Silk et al., 2014; Raufelder et al., 2021). Due to the importance of peer support for students’ psychological well-being, several secondary schools have implemented peer-based initiatives (Houlston et al., 2009). Within the context of mental health care, peer-to-peer support has been introduced as a promising way to enhance mental and physical well-being (Naslund et al., 2016).

However, no significant relationship was found between peer support and self-worth or physical well-being. Thus far, literature on bullying suggests that students’ physical well-being and self-worth are negatively impacted by peer rejection (Rigby, 2000; Grills and Ollendick, 2002; Rivers et al., 2009; Arslan et al., 2021). However, this link cannot be established in this study with respect to peer support, as peer support or the absence of peer support does not seem to be as relevant as the experience of peer victimization (cf. Raufelder et al., 2021). Among this age cohort, the function of peers seems to be focused on the psychological well-being of students, while teacher support becomes relevant for students’ self-worth and physical well-being. It seems that peer and teacher support compensate for each other with respect to students’ expression of self-worth, psychological well-being, and physical well-being, as students’ physical well-being and self-worth are related to teacher support.

Previous studies revealed teacher support to be related to students’ physical health with respect to their school exhaustion (Hoferichter and Raufelder, 2021) and school burnout (Meylan et al., 2015; Moots, 2019). Thus far, limited research has been conducted on teacher support and students’ self-worth, while results have been mixed (Riesen and Porath, 2004; Ozier, 2008; Zhang et al., 2021). However, the current study reveals that teacher support is related to students’ self-worth, which may be explained by the role teachers play in evaluating students’ competencies and academic development. In particular, teachers give frequent feedback—consciously and unconsciously—with respect to a student’s behavior and school performance, communicating their approval to the student (Hattie and Timperley, 2007), which, in turn, may be associated with student’s self-worth. This argument is supported by a neurobiological study which found that during an fMRI task, teacher appraisals, but not peer or self-referential appraisals, were linked to students’ academic self-concept (Golde et al., 2019). In fact, studies indicate that how students evaluate their abilities and themselves as people depends in part on the support and feedback of their teachers (Möller and Köller, 2001a,b; Skaalvik and Skaalvik, 2002; Möller et al., 2009; Kulakow and Hoferichter, 2021). In this regard, Booth and Gerard (2011) suggest teachers should use thorough feedback to improve students’ sense of their own abilities and provide opportunities to make them feel proud of their success. In a similar vein, Kulakow and Hoferichter (2021) recommend providing opportunities for students to experience competence. This can be achieved by considering students’ learning levels when designing tasks to avoid overtaxing students.

Although previous results indicate that teacher support is related to students’ psychological well-being (Tennant et al., 2014), the current study did not confirm these findings. This discrepancy may be explained by the general change in social relationships during adolescence. Within the investigated age group of middle school students, teacher-student relationships may be important when it comes to instructional support—which explains the positive link between teacher support and students’ self-worth—but not for socio-emotional support, which is related to psychological well-being (Hoferichter and Raufelder, 2021).

Hence, each source of support relates differently to students’ psychological and physical well-being as well as self-worth. This finding hints to the varying role of parents, teachers, and peers as socialization agents for middle school students. In detail, parents act as fundamental socialization agents, as parent-child interactions impact how children, respectively, students think about and value themselves, regulate their emotions, interact with others, view the world according to norms and values (Maccoby, 1994; Eisenberg et al., 1998), which may explain why parental support is associated with all three dimensions of well-being.

Considering the role of peers, whose importance peaks during the period of middle school, they contribute to students’ identity development (Ragelienė, 2016), by sharing common interests, values, and engaging in activities within the group. Being part of a peer group meets the basic psychological needs of belonging to a group and experiencing mutual trust, which explains why peer support is related to psychological well-being of students.

With respect to teachers, their interaction with middle school students is limited to the school context in which teachers commonly provide instructional support, praise and recognition as well as socio-emotional support with the aim to help students development academically (Hoferichter and Raufelder, 2021). Teachers’ focus on students’ academic development may explain why teacher support relates to students’ self-worth and physical well-being. Thereby, self-worth may be linked to students’ academic success and physical well-being may be linked to how students handle school-related stress which is prevalent during middle school (Hoferichter et al., 2021).

However, as the nature of the study is cross-sectional, it may be that students who have high well-being enjoy their relationships with parents, peers, and teachers more or they are more successful in maintaining social relationships with agents from their immediate environment and as such receive their support. In fact, individuals who feel happy and content have better social relationships than their less happy peers (Lyubomirsky, 2007). People who feel well, i.e., think positively about themselves, feel mentally and physically well, may be perceived as more attractive which increases the chance of initiating and maintaining social relationships with them. As people tend to share positive (and negative) events with each other (Gable et al., 2004), people with high well-being may experience more positive happenings which they share with others who in turn enjoy their positive view of life.

As such, the study results underline the ecological systems model (Bronfenbrenner, 1979) and the conceptual model of well-being in schools (Konu and Rimpelä, 2002), emphasizing that parent-child, student-student, and student-teacher relationships contribute to the well-being of students and thereby present differentiated resources for the specific dimensions of well-being (cf. Cohen and Wills, 1985; Hobfoll et al., 1990).

With respect to school form, the results indicate that students who attend higher-track schools reported higher levels of self-worth than those who attend lower-track and mixed-track schools, which also has been found by Van Houtte et al. (2012). The current study was conducted in Germany, whose educational system is characterized by early ability tracking. In most cases, students attend lower-track schools due to a lack of academic success often related to a recommendation from the teacher to attend lower-track schools after elementary school, as well as parental choice. It stands to reason that students who do not qualify to attend higher-track schools feel inferior, stigmatized and deprived, which is reflected by their relative low self-worth.

Considering gender differences, the current study reveals that boys reported higher levels of self-worth, psychological well-being, and physical well-being compared to girls. Some research suggests that self-worth develops differently for boys and girls as a consequence of social interactions and experiences related to the self. While females tend to integrate others into their self-schema, males rather see others as distinct, not being part of their self-schema (Josephs et al., 1992), which in turn impacts how they think about themselves. However, these gender differences primarily have been detected in Western oriented cultures which suggests that self-worth may be a result of how males and females are socialized (Bleidorn et al., 2016), underlining the impact of socialization agents such as parents, teachers, and peers who contribute to gender (non-)sensitive socialization processes.

With respect to psychological well-being, various studies indicate that women report lower psychological well-being compared to men (Gómez-Baya et al., 2018; Gómez-López et al., 2019). However, if women experience a satisfaction of their basic needs (e.g., autonomy, competence, and relatedness), they tend to rate their psychological well-being higher (Gómez-Baya et al., 2018). Addressing basic needs within the school context may give female students the opportunity to enhance their psychological well-being. Furthermore, girls may depict lower physical well-being compared to boys as they are more likely to be concerned and critical about their body image, which girls monitor more compared to their male counterparts (Salomon and Brown, 2019).

## Strengths, Limitations, and Future Direction

It is commonly recognized that parents, peers, and teachers present a significant context for students’ well-being. By investigating the relative association between parental, peer, and teacher support with students’ well-being, this study takes a detailed view on the complex nature of social relationships. In particular, potential protective factors are identified that stabilize and may contribute to school students’ self-worth, psychological and physical well-being during a period of increased vulnerability accompanied by puberty, environmental changes, and increased pressure to perform. The present detailed and systematic analysis calls for continuing longitudinal and interventional studies that may be able to offer conclusive evidence about causal relationships between sources of support and various dimensions of well-being. The present investigation provides a basis for the planning and shaping of support with the aim to enhance students’ well-being and enable school students to flourish. This also leads to one limitation of the study: The study’s design is cross-sectional and therefore does not allow for causal conclusions. As such, students were asked about their perception of support and how they evaluate their well-being. Although students’ self-perception was of interest for the study, further investigations may consider multi-perspective ratings, for example, from parents, teachers, and peers, with respect to the variables of interest. Future studies may also focus on girls’ and boys’ perceptions of social support in relation to well-being to pinpoint gender-specific nuances. Person-oriented approaches (e.g., profile analysis) would allow researchers to derive implications for specific student groups (e.g., students with migrant backgrounds, students with disabilities, or students diagnosed with behavioral or mental health problems) to enhance their well-being.

## Data Availability Statement

The raw data supporting the conclusions of this article will be made available by the authors, without undue reservation.

## Ethics Statement

The studies involving human participants were reviewed and approved by the Ministry of Education, Science and Culture. Written informed consent to participate in this study was provided by the participants’ legal guardian/next of kin.

## Author Contributions

FH designed the study, collected the data, developed the theoretical framework, and wrote main parts of the manuscript. MH and SK conducted the statistical analyses and wrote the analysis, and results section. All authors contributed to the article and approved the submitted version.

## Conflict of Interest

The authors declare that the research was conducted in the absence of any commercial or financial relationships that could be construed as a potential conflict of interest.

## Publisher’s Note

All claims expressed in this article are solely those of the authors and do not necessarily represent those of their affiliated organizations, or those of the publisher, the editors and the reviewers. Any product that may be evaluated in this article, or claim that may be made by its manufacturer, is not guaranteed or endorsed by the publisher.
